# Supplementary material for: Peroxisome proliferator‐activated receptor gamma (PPARγ) regulates lactase expression and activity in the gut
Source: EMBO Mol Med. 2017 Sep 25;9(11):1471–81. doi: 10.15252/emmm.201707795 (PMC5666307; doi:10.15252/emmm.201707795)
Supplement: Supplementary file 1 — Appendix [file EMMM-9-1471-s001.pdf]

Appendix

Peroxisome proliferator-activated receptor gamma (PPARγ) regulates lactase expression and activity in the gut

Mathurin Fumery[1,2,9,10], Silvia Speca[1,2,10], Audrey Langlois[1,2], Anne-Marie Davila[4], Caroline Dubuquoy[5], Marta Grauso[4], Anthony Martin Mena[1,2], Martin Figeac[6], Daniel Metzger[7], Christel Rousseaux[5], Jean-Frederic Colombel[8], Laurent Dubuquoy[1,2], Pierre Desreumaux[1,2,3], Benjamin Bertin[1,2]

[1]Univ. Lille, U995 - LIRIC - Lille Inflammation Research International Center, F-59000 Lille, France
[2]Inserm, U995, F-59000 Lille, France
[3]CHU Lille, Service des Maladies de l'Appareil Digestif et de la Nutrition, Hôpital Claude Huriez, F-59037 Lille, France
[4]UMR0914 Institut National de la Recherche Agronomique/AgroParisTech, Université Paris-Saclay, 75005 Paris, France
[5]Intestinal Biotech Development, F-59000 Lille, France
[6]Functional and Structural Genomic Platform, Université de Lille, Lille, France
[7]Institut de Génétique et de Biologie Moléculaire et Cellulaire, CNRS UMR7104/INSERM U964/Université de Strasbourg, Collège de France, Paris, France
[8]The Henry D. Janowitz Division of Gastroenterology, Icahn School of Medicine at Mount Sinai, New York, New York.
[9]Service d'Hépatogastroentérologie, Centre Hospitalier Universitaire d'Amiens, Université de Picardie Jules Verne, Amiens, France
[10]These authors contributed equally to the work

Table of content Page

Appendix Figure S1: GED and pioglitazone stimulations do not modify the glucose uptake capacity of Caco-2 cells. 3

Appendix Figure S2: In silico identification of PPRE in the LCT gene promoter. 4

Appendix Figure S3: ChIP and reporter gene assays from Caco-2 cells stimulated with pioglitazone 5

Appendix Figure S4: Caco-2 colorectal cell line knock-down for PPARγ. 6

Appendix Figure S5: Detection of LCT and PPARγ proteins in the human duodenum. 7

Appendix Figure S6: stool consistency score and weight loss in rats fed with lactose-enriched diets.. 8

Appendix Table S1: composition of control and lactose-enriched diets. 9

Appendix Table S2: Oligonucleotides used in the study 10

Additional references 11

Appendix Figure S1

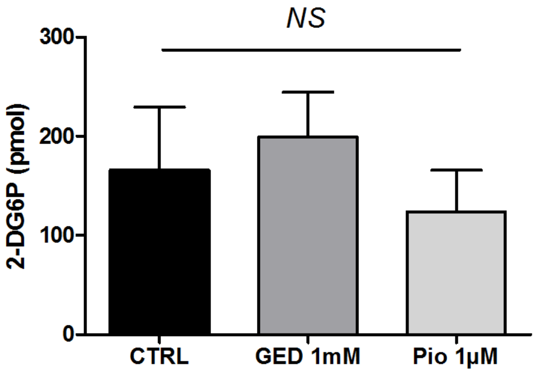

Appendix Figure S1: GED and pioglitazone stimulations do not modify the glucose uptake capacity of Caco-2 cells. Caco-2 cells were stimulated for 24h with 1mM GED or 1µM Pio and glucose uptake was then assessed. Glucose uptake was measured using the glucose analogue, 2-deoxyglucose, which is taken up by cells and phosphorylated to 2-DG6P. 2-DG6P cannot be further metabolized and accumulates in the cells, and the 2-DG6P level is directly proportional to glucose uptake by cells. The result is expressed as the amount of 2-DG6P (pmol) measured in the cells. Data are expressed as mean ± SD (n=6). NS, not significant (two-tailed nonparametric Mann-Whitney tes).

Appendix Figure S2

A

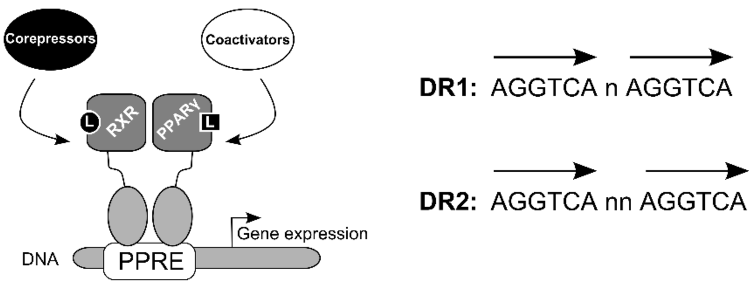

B

```
GTAATTTATTTTACTTCTGTGTCCTAAGGGTAATTTCTCAGGATTGTTTTCAAATTGCTTTTTTAGGGGAAATAGGTCAT
TTGCTATATTACAAGCAATCCCCAAATTTTATGGTCTTCCAGGAAAAGTTATTACCGTTTATGATACTAACAGTTCCTGA
GACTTAGCTATGATCAGTATGTTCATGAGGTGGAGCAGTTCCTGTGTTGCAGCTTTTAACAACAGATGGCATTCATTAAA
TCACAAAGTATGTTAAAGGTCACAAAAGCAAAATAACTGTCTGAGGCTAAGGCCCACGTGGGACAGTCTAATACCCATGA
GTACTCAACTTGCCTTGATGTCTGAGCTTTCCAGTGCAATGTGAATTTGAGCAGCCAGAAATCTATTAGTAGAAAGCAAG
ACAGATTAATATAGGTTAAAACAATGATTTAAATATGTTTCTCCCAATAATTATCTCTTTCCCTGGAATCAACTTGTATG
AAACCTTGTCAAAATGTACTCCACAAGTATGTACAATTAAGTATTTTAAAAATAAATGGCAAACATTAAAAACAAGAGTG
AATACTCAAGTAGATTTGTCATGGGATTTTTATAAGAAGACTGGTATCAGGTAATGTATCTTTAAAGACTAGGCTGCTCT
GCTGACGTAGTAACCATTTTTTATTCCTTTACTTTCCTAATAGCCTTGCTTTCACTTAAGAAAAAAAAAGGCTAGGCACG
GTGGCTCACGCCTGTAATCCCAGCACTTTGGGAGGCCAAGGTGAGTAGATCACCTGAGGTCAGGAGTTCGAGACAAGCCT
GGCCAACGTGGAGAAACCTGTCTCTACTAAAAGTACAAAAATTAGCCAGGCATGGTGGTGGGCACCTGTAATCCCAGCT
ACTCAGGAGGTTGAGGCAGGAGAATTGCTTGAACCCAAGAGGTGGAAGTTGCAGTGAGCTGAGATCATGCCACTGCCCTC
CAGCTTGGGCGGCAACAACAACAACAAAAAAAGATTCCCAGGCTTCACCCTCTACAGTCTATGGAAAGTCATGGGAGTCT
ATTAAAAAAAAACAGACACTATAAACCAAATTAAAAGATGGGAAAATTTTTCTATCACGTTTTAATATGTGATATCCAAA
CTCCCATTAAGAATTTTTATATCAGTAATAAGATAAAACTCAGAAGAAAAATGCCCAATTAACAAGAAGAGCTTCACCTC
TTAAATAATCAGAAAAAGAAAAGCACATAATATTTACGTCAAAGTATCAAAAATGTAAAAGTTTTATAATAGGCATTAGC
AAGGCAGACGGATACAGGCACACATCTAACTCTATGTAGGATTTAACTTGGAAGACAATTTGCATTTCAATCAATCAAAA
ACCATTTTTTTTTTTGAGATGATCTCCCTGTGTTGCCCAGACTGGCCTCAAGCTCCTGGGCTTGAGCAGTCCTACAGTCT
GAGCCTCCAAAAGTACTGGCAAAACAGGCGTGATTGACCATGCCCAGCCTGTATATAAGGATATTCATTTGCAGCATTGT
TTGCAACACAAAAATGTAAAACCAACCGGTGTTTAATATGCAAGATGTTAATTTATGGTACATTCTTGCTGTGGAAAGCT
ATGCATCTGTTAGAGGTGGGTCTATATGTTCTGATACAGGCAGAACTCTAAGAGCTATTAAGTGAAGAAAAGGCTGAAAA
TACATATGGCATAATTCCATATATGTTAAATTGTTCTATATTTTAAATGTTATACACGTTTCCATTTGTATTTACGTAAT
AATAATGATCCCTGAGCTGTACTGTTGTATACATGCTACAAGAAAGACCCATTTAATCCCCACAGCCTATGATGAATTAT
CCACATTCTACAGGTGACAAAATAGAGGCACAAAGTTAAGTAATTTTTGTCAGGTGAGATTTAAACCCAGGCATTCTGAC
TCCTGTATAACCATTAAGATATGCAGAGAAAGAAAACTGGAAAGATACATATTGCTGAAGATACTTATTATAGGAAGAGG
AGGGGGGAGGGTGAAGGAATTTGCAAGTTTTTCATAGATGTTTCCATATTGTTTGAATCTCTTACAAAATATGTTCAGCA
TATTTTTAAAGAGAAAATTTGGGGCAAAATACTTATTTTTGTATTATGTAAACAAATTTTAAAATAATGTGTGGCTGGGT
GCGCTGGCTCACACCTGTAATCCCAACACTTTAGGAGGCTGAGGCAAGAGGATTGCTTGAGCCCAGGAGTTCAAGACCAG
CCTGGGTGACATGGCAAAACTCCATCTCTACTAAAAATACAAAAAATTAGCCAGTCGTGGTGGCGCACACCTATGGTCCC
ACCTACCCAGGATGCTGAGATGGGAGCATCACTTGAGCCCAGGAAGTCAAGGCTGCAGCAAGCTGTGATCGCACCACTGC
ACTCCCACCTGGGCAACAGAGTGAGACCCGGTCACCAAAAAACAAAAAAAACAAAAAAAATTGGTAATCGTTTTCTTCAG
ACATTTTCCGGGTTCCTCTGCTTAACTTGTATAGGAAGTCTGAGGTTTTGTGTTGGTCTTTACCTTTTTTTTTTTTTTTT
TTTTTTTAAGATGGAGTCTCATTCTGTTGCCCAGGCTGGAGTGCAGTGGCATGATCTTGGCTCCTGCAACCTCCGCCTCC
TGGGTTCAAGTGATTCTCCTGCCTCAGCCTCCTGAGTAGCCGGGACTACAGGCGCATGCCACGATGCCTGGCTAATTTTT
TGTATTTTTAGTAGAGATGGGGTTTCACCATGTTAGCTAGGACGGTCTCGATCTCCTGACCTCGTGATCCGCCCACCTCG
GCCTCCCAAAGTGCTGGAATTACAGGTGTGAGCCACCACGCCCGGCCCTGATCTTTACATTTTTAAATATTGCATTAGTG
AACCGTGTACTGATTTTGTGATCATAGATAACCCAGTTAAATATTAAGTCTTAATTATCACTTAGTATTTTACAACCTCA
GTTGCAGTTATAAAGTAAGGGTTCCACATACCTCCTAACAgttcctagaaaa
```

Appendix Figure S2: *In silico* identification of PPRE in the LCT gene promoter. **(A)** PPARγ heterodimerizes with the 9-cis-retinoic acid receptor (RXR) to bind to response elements (PPREs) located in target genes to activate transcription. PPARγ-RXR is a permissive heterodimer, as the transcriptional activity of the dimeric complex can be activated by both PPARγ and RXR agonists. PPARγ-RXR heterodimer mainly recognizes PPRE composed of a direct repetition of the consensus sequence AGGTCA separated by one (DR1) or two (DR2) nucleotide(s). **(B)** We used bioinformatics tools to find PPRE (DR1 and DR2) in the human LCT promoter gene (3,000 bp upstream to the transcription start point). Three different programmes were used: NUBIScan (Podvinec et al, 2002), PPRE Search (http://www.classicrus.com/PPRE/) (Venkatachalam et al, 2009) and MatInspector (Genomatix; http://www.genomatix.de). The putative DR1 identified in the 3,000 bp sequence of the LCT gene promoter are denoted in red and underlined and the putative DR2 identified are denoted in blue and underlined. The nucleotides shown in green and underlined denote potential TATA box. Italic nucleotides in purple indicate the location of primers 8a and 8b used in the ChIP experiment.

Appendix Figure S3

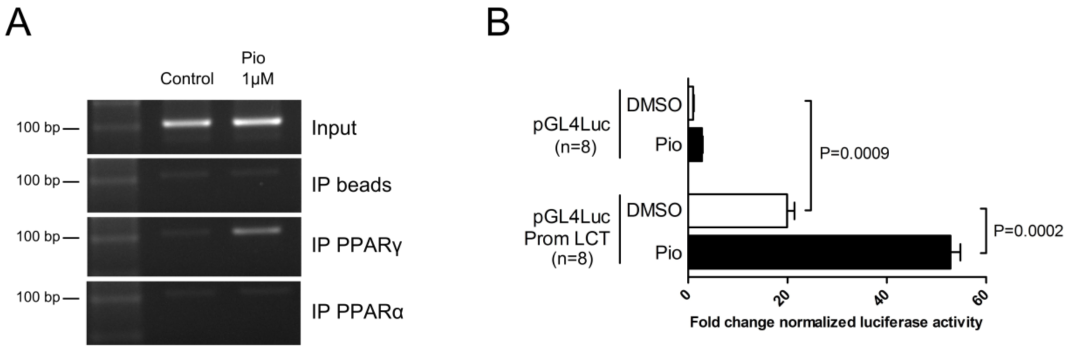

Appendix Figure S3: ChIP and reporter gene assays from Caco-2 cells stimulated with pioglitazone. (A) PCR amplification of the 8a-8b fragment in ChIP assay from Caco-2 cells stimulated with Pio 1μM. **(B)** Luciferase gene reporter assay in Caco-2 cells transfected with pGL4Luc-PromLCT and stimulated with Pio 1μM. Cells transfected with empty pGL4Luc were used as control. Results represent the fold change of luciferase activity normalized for protein content (pGL4luc-DMSO condition defined as 1). Data are expressed as mean ± SEM (n=8 for each condition; two-tailed nonparametric Mann-Whitney test).

Appendix Figure S4

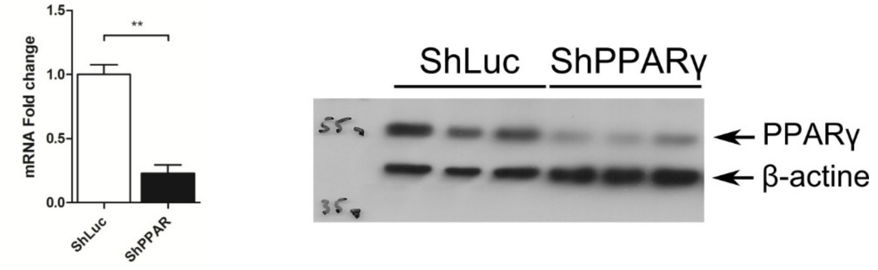

Appendix Figure S4: Caco-2 colorectal cell line knock-down for PPARγ. Caco-2 colorectal cell line knock-down for PPARγ (ShPPAR) expressed significantly fewer PPARγ transcripts and protein levels compared to ShLuc control cells. Quantitative expression of mRNA was assessed by qRT-PCR. Results represent the mean values of sextuplicate ± SD of the fold change of PPARγ expression normalized to the β-actin expression level. The expression level measured in ShLuc cells (arbitrarily defined as one) was used as reference. Protein level was assessed by Western blot. **P=0.0022 (nonparametric Mann-Whitney Test). These cell lines have already been described in (Bouguen et al, 2015).

Appendix Figure S5

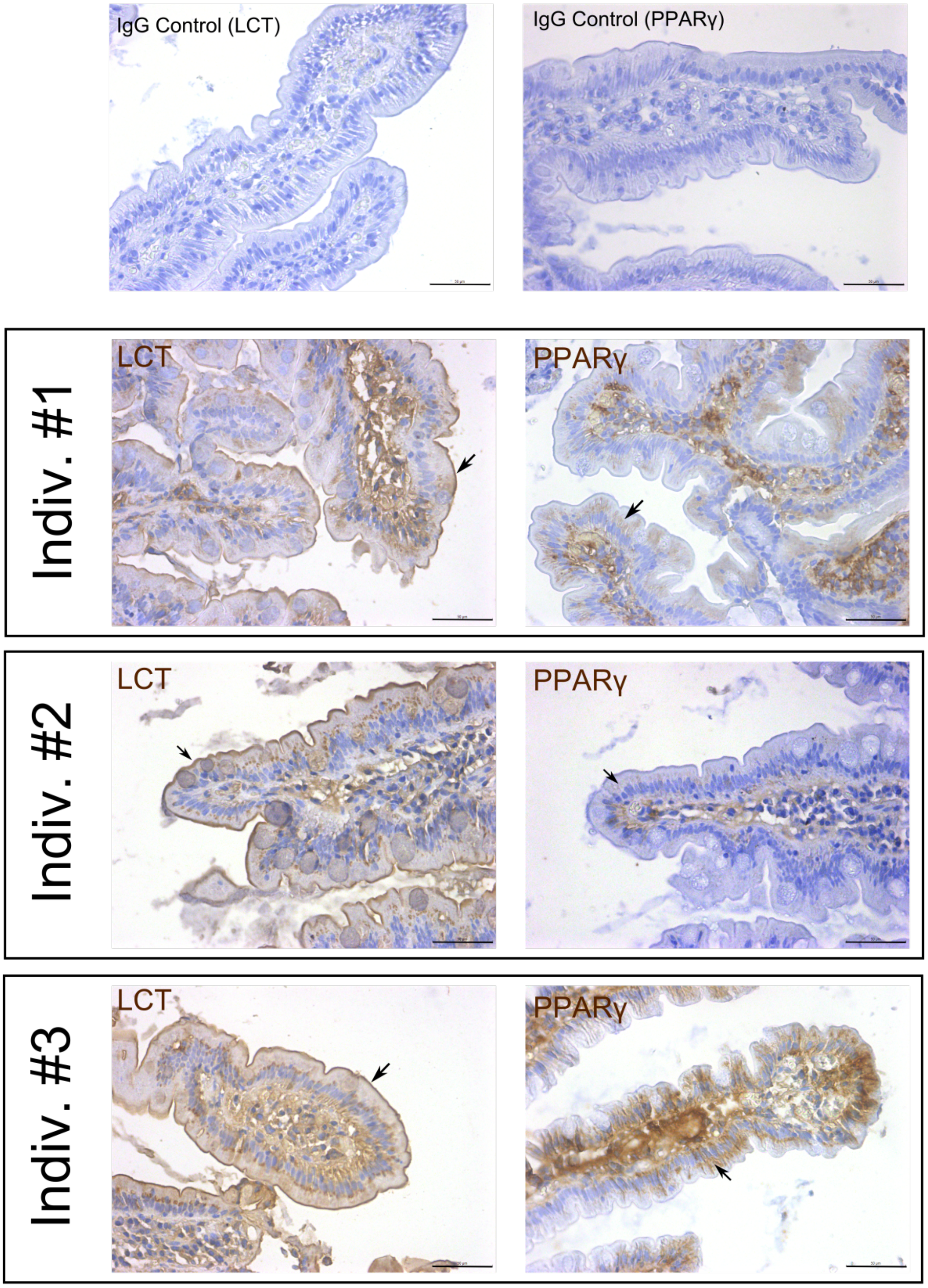

Appendix Figure S5: Detection of LCT and PPARγ proteins in the human duodenum. Immunohistochemical staining for LCT (left) and PPARγ (right) proteins in human duodenal specimens. Arrows indicate specific staining. Magnification X40. Scale bar: 50 µm.

Appendix Figure S6

A

Score	Appearance	Description
1	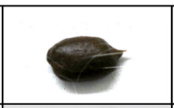	Normal feces<br>Separate hard lumps, like nuts
2	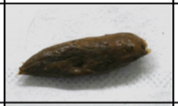	Round shapes with firm consistency
3	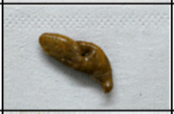	Like a well defined sausage,<br>but still muschy
4	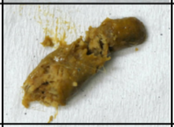	Soft blobs with clear cut edges
5	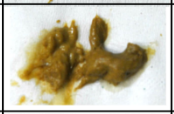	Musty/Fluffy stool with ragged edges
6	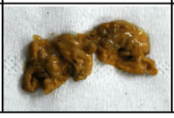	Watery Stool with no solid pieces

B

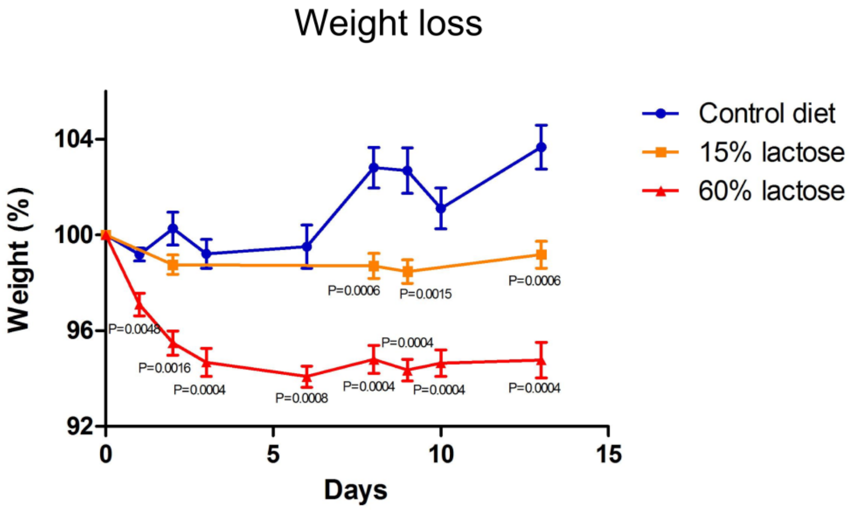

Appendix Figure S6: stool consistency score and weight loss in rats fed with lactose-enriched diets. (A) Stool consistency score **(B)** Weaned rats (9<n<10) were fed with lactose-enriched diets (15% lactose or 60% lactose as indicated) or control isocaloric diet and animals were monitored for 13 days. Results represent the time-course of body weight variation (mean values ± SEM). P values between lactose groups and control diet are indicated.

Appendix Table S1

Product No.		60 % Glc – lactose *customized*	45 % Glc 15 % Lac *customized*	– Glc 60 % Lac *customized*
Casein	%	20,000	20,000	20,000
Corn starch, pre-gelat.	%	2,600	2,600	2,600
Maltodextrin	%	——	——	——
Sucrose	%	——	——	——
**Glucose / Dextrose**	%	**60,000**	**45,000**	——
Lactose	%	——	15,000	60,000
Cellulose	%	5,000	5,000	5,000
L-Cystine	%	0,200	0,200	0,200
Vitamin premixture *	%	1,000	1,000	1,000
Mineral & trace element premix	%	6,000	6,000	6,000
Choline Cl	%	0,200	0,200	0,200
Soybean oil	%	5,000	5,000	5,000
Crude protein (= N x 6.25)	%	17,7	17,7	17,7
Crude fat	%	5,1	5,1	5,1
Crude fiber	%	5,0	5,0	5,0
Crude ash	%	5,3	5,3	5,3
Starch	%	2,5	2,5	2,5
Dextrins	%	——	——	——
Sugar (total)	%	60,7	60,7	60,7
Carbohydrates	%	64,3	64,3	64,3
ME (Atwater)	MJ/kg	15,7	15,7	15,7
kcal% Protein		19	19	19
kcal% Fat		12	12	12
**kcal% CHO**		**69**	**69**	**69**

Appendix Table S1: composition of control and lactose-enriched diets. Customized rodent chows were obtained from Ssniff Spezialdiäten GmbH (Soest, Germany).

Appendix Table S2

Gene	Sens oligonucleotides	Anti-sens oligonucleotides
**Human Lactase**	5'-GCGGCAGAAGCTGAGTAAAT-3'	5'-AAGGCTGAAGAGCAGACTGG-3'
**Human GAPDH**	5'-GACACCCACTCCTCCACCTTT-3'	5'-TTGCTGTAGCCAAATTCGTTGT-3'
**Human Sucrase-Isomaltase**	5'-GGATGGTCACAGATGAAACCT-3'	5'-TCCTCCCCATCGTCCACTA-3'
**Human Maltase-glucoamylase**	5'-GACATTGGCTGGGAGACAAC-3'	5'-CTCCCGTATAGGATATGCCG-3'
**Human PPARgamma**	5'-AGGCCATTTTCTCAAACGAG-3'	5'-CCATTACGGAGAGATCCACG-3'
**Mouse Lactase**	5'-GCATGCGCCAGCCATCTCTGA-3'	5'-CACAATGCCCACGCGTCCCT-3'
**Mouse beta-Actin**	5'-TGGAATCCTGTGGCATCCATGAAAC-3'	5'-TAAAACGCAGCTCAGTAACAGTCCG-3
**Rat Lactase**	5'-CCATATTGAGCTGTTGCCGC-3'	5'-GCCCTTCCTTTCCTGTGTCA-3'
**Rat GAPDH**	5'-CTGTTCTAGAGACAGCCGCATCT-3'	5'-ACACCGACCTTCACCATCTTG-3'
**Rat PPARgamma**	5'-TGCACTGCCTATGAGCACTT-3'	5'-TGTCAAAGGAATGCGAG-3'
**ChIP 8a-8b**	5'-CACCATGTTAGCTAGGACGGTCTC-3'	5'-CGTGGTGGCTCACACCTGTAATTCC-3'
**Hs-Prom-0.3Kb**	5'-CTCTCGAGGCCGGGACTACAGGCGCATGCCAC-3'	5'-CTAAGCTTGTTAGGAGGTATGTGGAACCCTTAC-3'
**C/T$_{13910}$ polymorphism**	5'-GGACATACTAGAATTCACTGCAA-3'	5'-GGTTGAAGCGAAGATGGGACG-3'
**G/A$_{22018}$ polymorphism**	5'-CAGAGCTGTCTACACCAGTGGTA-3'	5'-AGCTGGGACCACAAGCACCCGCCACCATGCGCGGCTAAT-3'
**0.3kb Mut**	5'-GTCTCGATCTCCTGAAAACGTGATCCGCCCACCTC-3'	5'-GAGGTGGGCGGATCACGTTTTCAGGAGATCGAGAC-3'
**0.3kb Del**	5'-GTCTCGATCTCCTGACGTGATCCGCCCACCTC-3'	5'-GAGGTGGGCGGATCACGTCAGGAGATCGAGAC-3'

Appendix Table S2: Oligonucleotides used in the study

ADDITIONAL REFERENCES

Bouguen G, Langlois A, Djouina M, Branche J, Koriche D, Dewaeles E, Mongy A, Auwerx J, Colombel JF, Desreumaux P et al (2015) Intestinal steroidogenesis controls PPARgamma expression in the colon and is impaired during ulcerative colitis. *Gut* 64**:** 901-910

Podvinec M, Kaufmann MR, Handschin C, Meyer UA (2002) NUBIScan, an in silico approach for prediction of nuclear receptor response elements. *Mol Endocrinol* 16**:** 1269-1279

Venkatachalam G, Kumar AP, Yue LS, Pervaiz S, Clement MV, Sakharkar MK (2009) Computational identification and experimental validation of PPRE motifs in NHE1 and MnSOD genes of human. *BMC Genomics* 10 Suppl 3**:** S5
