# Supplementary material for: Peroxisome proliferator‐activated receptor gamma (PPARγ) regulates lactase expression and activity in the gut
Source: EMBO Mol Med. 2017 Sep 25;9(11):1471–81. doi: 10.15252/emmm.201707795 (PMC5666307; doi:10.15252/emmm.201707795)
Supplement: Supplementary file 2 — Expanded View Figures PDF [file EMMM-9-1471-s002.pdf]

Expanded View Figures

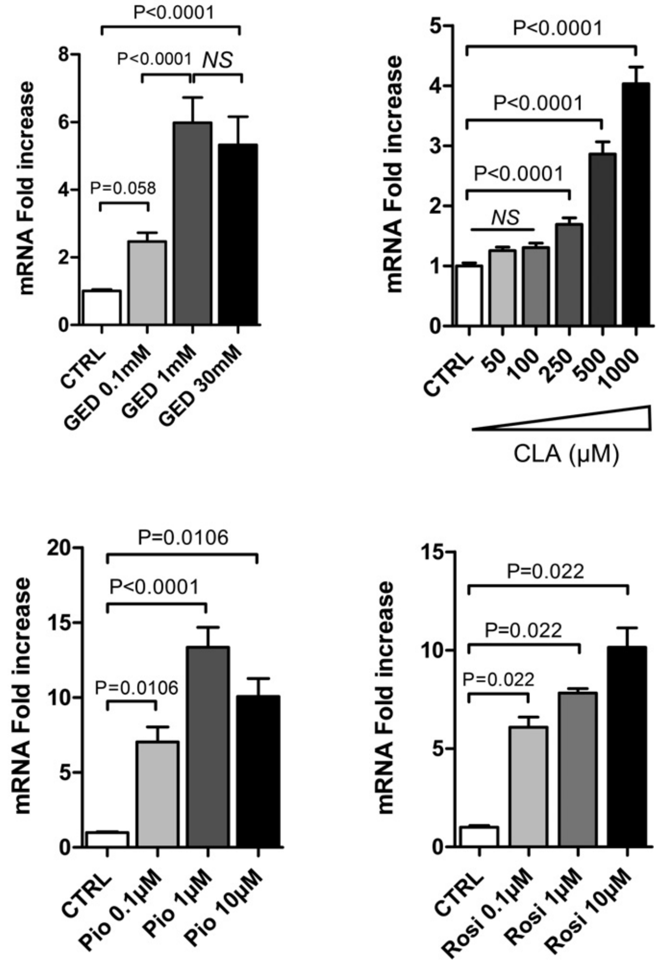

Figure EV1. Dose effect of GED, CLA, pioglitazone, and rosiglitazone on LCT expression in Caco-2 cells.

Caco-2 cells were stimulated with various concentrations of each PPARγ agonist for 24 h as indicated. LCT gene expression was determined by qRT–PCR of corresponding reverse-transcribed mRNA. Results represent the mean ± SEM (two to three independent experiments in triplicate or sixplicate) of the fold change of LCT gene expression. The expression level measured in control cells, arbitrarily defined as one, was used as reference. NS, not significant. Statistical analysis: two-tailed nonparametric Mann–Whitney *U*-test.

 © 2017 The Authors

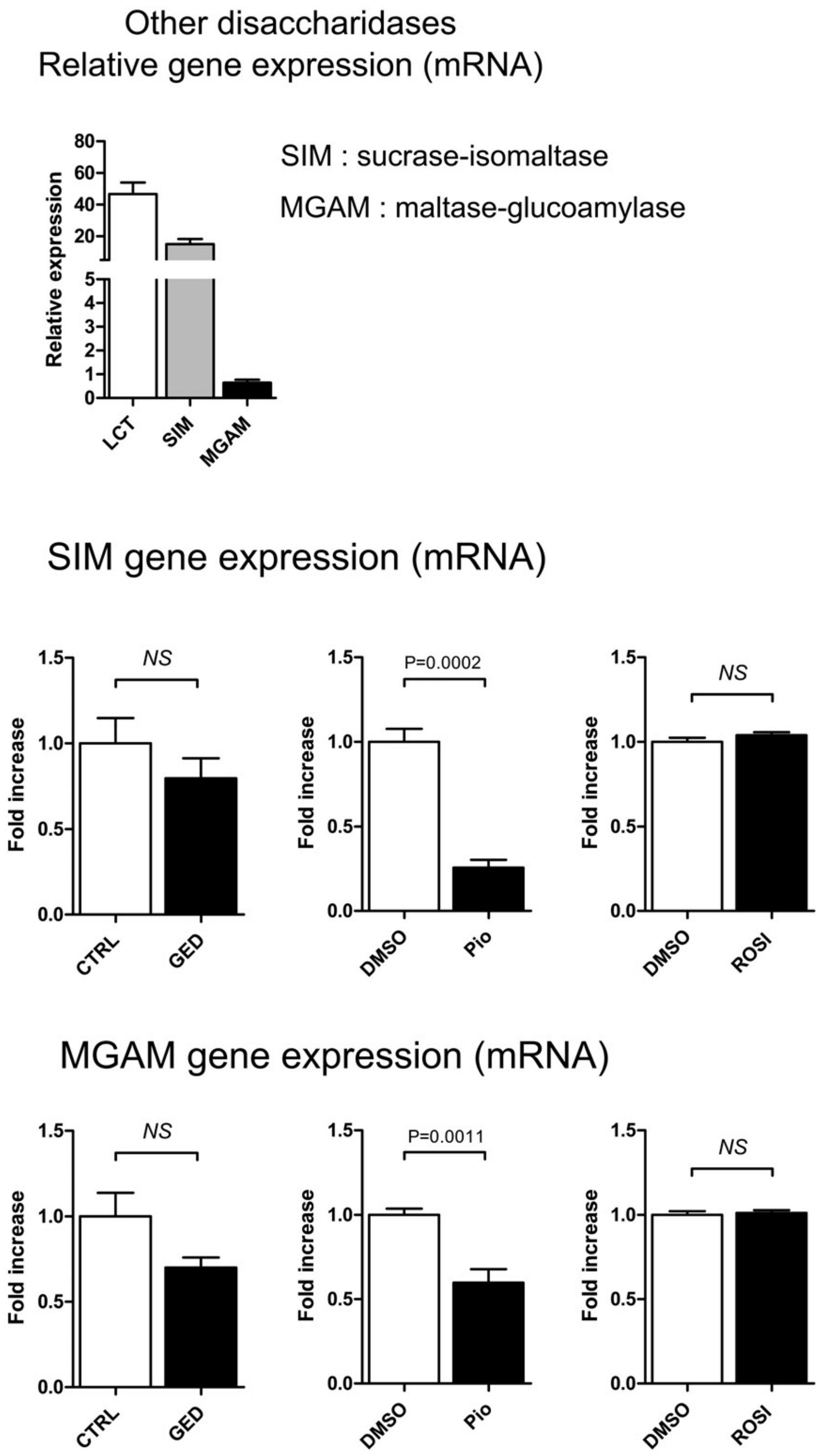

Figure EV2. GED, pioglitazone, and rosiglitazone do not induce upregulation of other disaccharidases expressed by Caco-2.

Relative expression of genes encoding sucrase-isomaltase (SIM) and maltase-glucoamylase (MGAM) compared to LCT in Caco-2 cells as determined by qPCR analysis of reverse-transcribed mRNA. qPCR analysis of SIM and MGAM mRNA expression in Caco-2 cells stimulated with 1 mM GED, 1 μM Pio, and 1 μM Rosi. Results represent the mean ± SEM (two independent experiments, $8 < n < 9$) of the fold change of expression of SIM and MGAM genes normalized to GAPDH level. The expression level measured in control cells, arbitrarily defined as one, was used as reference. NS, not significant. Statistical analysis: two-tailed nonparametric Mann–Whitney *U*-test.

© 2017 The Authors

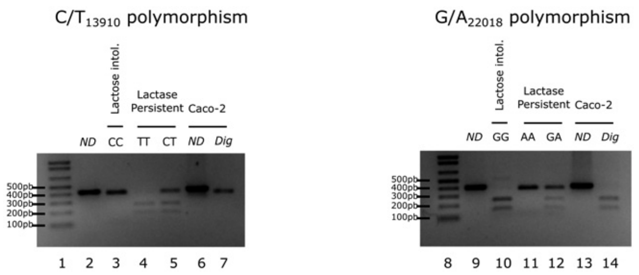

Figure EV3. LCT genotyping of polymorphisms C/T_{13910} and G/A_{22018} for Caco-2 cells.

DNA genomic fragments encompassing C/T_{13910} and G/A_{22018} nucleotides were amplified and digested as described in Matthews *et al* (2005). The digested PCR products were analyzed on agarose gel. Lanes 1 and 8 show molecular weight markers. Lanes 2 and 9 show non-digested (ND) PCR products. Lanes 3 and 10 show the CC_{13910} and GG_{22018} homozygous lactose intolerance genotypes, respectively. Lanes 4 and 11 show the TT_{13910} and AA_{22018} homozygous lactase persistent genotypes, respectively. Lanes 5 and 12 show the CT_{13910} and GA_{22018} heterozygous lactase persistent genotypes, respectively. Lanes 6 and 13 show non-digested PCR products from Caco-2 cells. Lanes 7 and 14 show digested PCR products displaying LCT genotyping polymorphisms C/T_{13910} and G/A_{22018} for Caco-2 cells. Migration profile reveals that Caco-2 cells possess the CC_{13910} (lane 7) and GG_{22018} (lane 14) homozygous lactose intolerance genotypes.

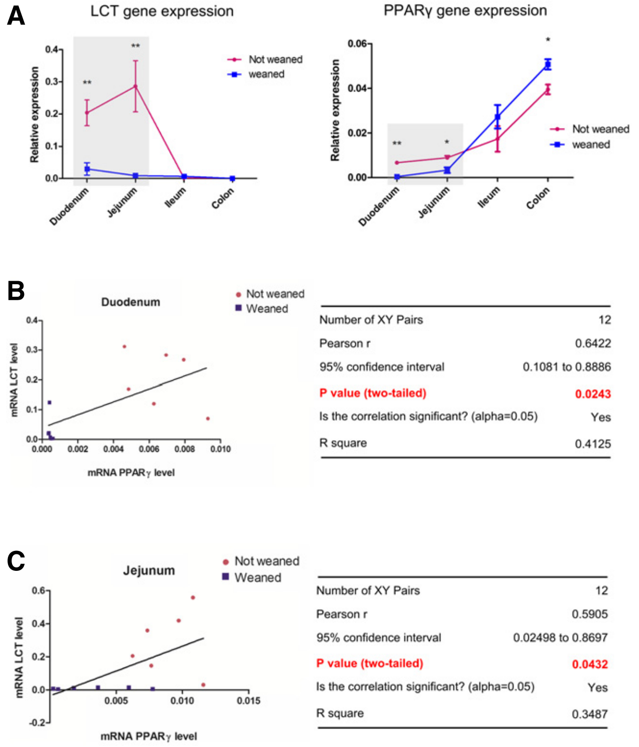

Figure EV4. LCT and PPARγ genes expression correlate in the rat duodenum and jejunum.

A Comparison of the LCT and PPARγ mRNA levels along the gut of "not weaned" and "weaned" rats. Gene expression level was determined by qPCR of corresponding mRNA. Results represent the mean ± SD of the relative expression normalized to GAPDH level (for each group $n = 6$). *$P < 0.05$, **$P < 0.01$. Statistical analysis: two-tailed nonparametric Mann–Whitney *U*-test.

B, C Correlation between the LCT mRNA and PPARγ mRNA levels in the duodenum and jejunum of rats (not weaned and weaned).

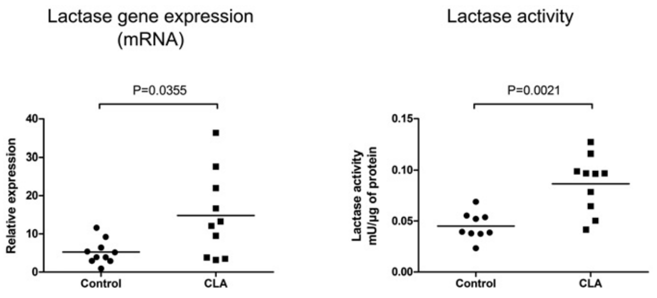

Figure EV5. LCT expression and activity in rats following CLA administration.

LCT gene expression (qPCR) and LCT activity were assessed in the proximal small intestine of weaned Sprague Dawley rats treated with oral CLA (200 mg/kg) for 5 days. Horizontal bars represent mean values ($n = 10$). Statistical analysis: two-tailed nonparametric Mann–Whitney *U*-test.

 © 2017 The Authors
