# Supplementary figures and images for: Peroxisome proliferator‐activated receptor gamma (PPARγ) regulates lactase expression and activity in the gut
Source: EMBO Mol Med. 2017 Sep 25;9(11):1471–81. doi: 10.15252/emmm.201707795 (PMC5666307; doi:10.15252/emmm.201707795)

SOURCE DATA FIGURE 1B

Magnification X40

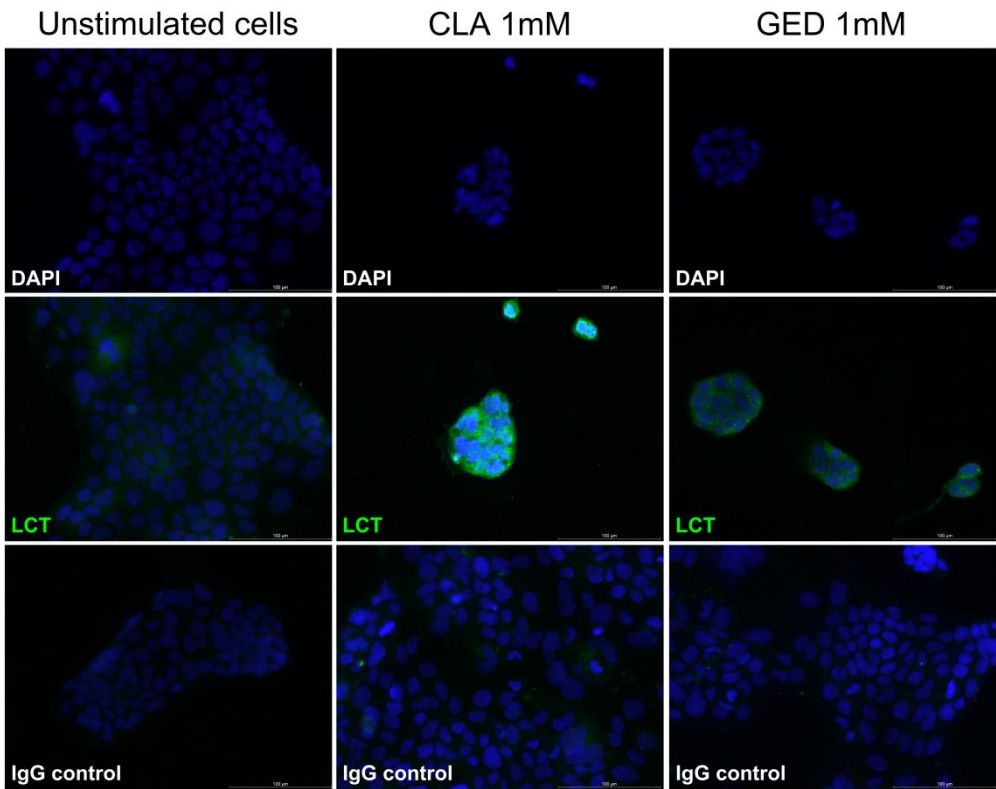

Magnification X40

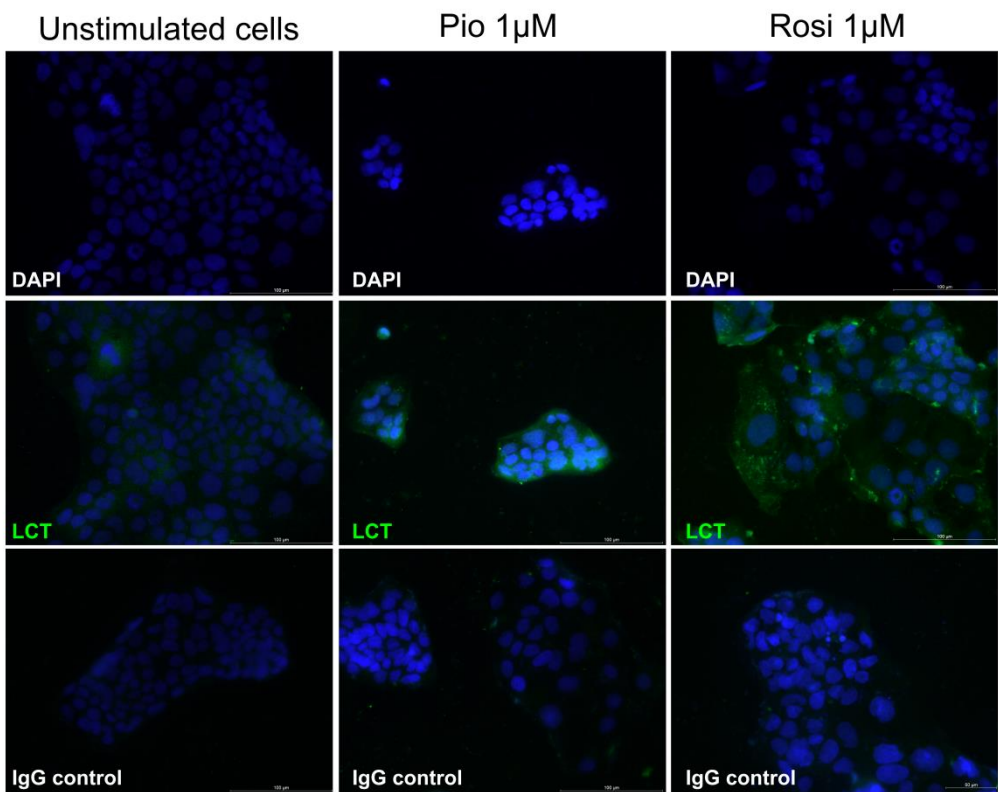

SOURCE DATA FIGURE 1C

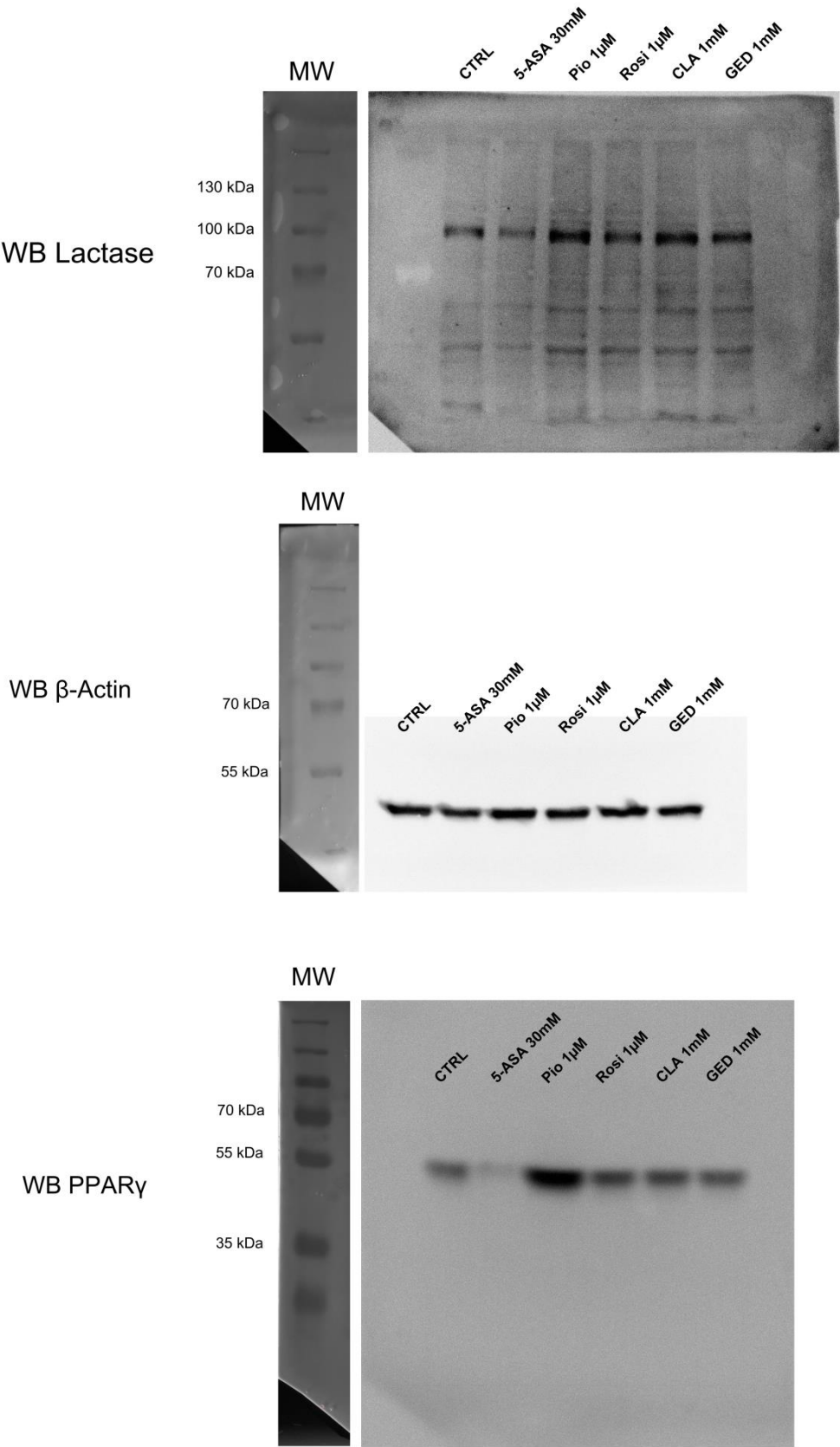

Supplement: Supplementary file 4 — Source Data for Figure 1 [file EMMM-9-1471-s003.pdf]
